# Localized adaptive evolution in VP1 drives antigenic divergence of feline calicivirus despite high sequence conservation

**DOI:** 10.1186/s13567-026-01778-y

**Published:** 2026-05-26

**Authors:** Yiqian Jiang, Yu Qi, Boli Ma, Faming Miao, Huixian Yue, Tianying Hou, Teng Chen, Shuchao Wang, Nan Li, Yanyan Zhang, Rongliang Hu, Shoufeng Zhang

**Affiliations:** 1 State Key Laboratory of Pathogen and Biosecurity, Changchun Veterinary Research Institute, Chinese Academy of Agricultural Sciences, Changchun, People’s Republic of China; 2 College of Life Sciences, Ningxia University, Yinchuan, People’s Republic of China; 3 College of Veterinary Medicine, Jilin Agricultural University, Changchun, People’s Republic of China

**Keywords:** Feline calicivirus, viral evolution, VP1 capsid protein, antigenic divergence, immune escape, localized adaptation

## Abstract

Feline calicivirus (FCV) evolves rapidly under immune pressure, yet the genetic basis of antigenic escape remains poorly understood, particularly when sequence divergence is limited. We isolated 13 FCV strains from cats in Jilin Province, China, between 2018 and 2024, and characterized their genetic and antigenic properties. Phylogenetic analysis of the major capsid protein VP1 revealed the co-circulation of multiple lineages, including isolates clustering within clades associated with virulent systemic disease (VSD) and grouping with strains previously identified in dogs. Cross-neutralization assays demonstrated that the vaccine-induced antiserum neutralized most isolates but exhibited markedly reduced neutralizing activity against JL1907 and JL2103, both of which were isolated from vaccinated cats. Notably, despite sharing 91.9% amino acid identity in VP1, JL2103 and JL2104 exhibited highly asymmetric cross-neutralization: Antiserum raised against JL2103 failed to neutralize JL2104, whereas antiserum raised against JL2104 potently neutralized JL2103. Comparative sequence analysis identified nonsynonymous substitutions concentrated in E region (the hypervariable region of VP1), particularly within predicted B cell epitopes and residues proximal to the feline junctional adhesion molecule A (fJAM-A) binding interface. Our findings indicate that minor amino acid substitutions at critical antigenic sites are sufficient to disrupt cross-neutralization, even among closely related FCV strains, thereby providing a mechanistic basis for antigenic escape under constrained genetic divergence.

**Supplementary Information:**

The online version contains supplementary material available at 10.1186/s13567-026-01778-y.

## Introduction

Feline calicivirus (FCV), a member of the *Vesivirus* genus within the Caliciviridae family, is a non-enveloped virus with an icosahedral capsid of 27–40 nm in diameter that encapsulates a single-stranded, positive-sense RNA genome [1]. The capsid exhibits *T* = 3 icosahedral symmetry and comprises 90 dimers of the major capsid protein [2]. The low fidelity of the virus-encoded RNA-dependent RNA polymerase endows FCV with high genetic plasticity, facilitating rapid adaptation to selective pressures [3]. Such a structure, a naked capsid without a lipid envelope, also contributes to pronounced environmental stability of FCV.

**Figure 1:**
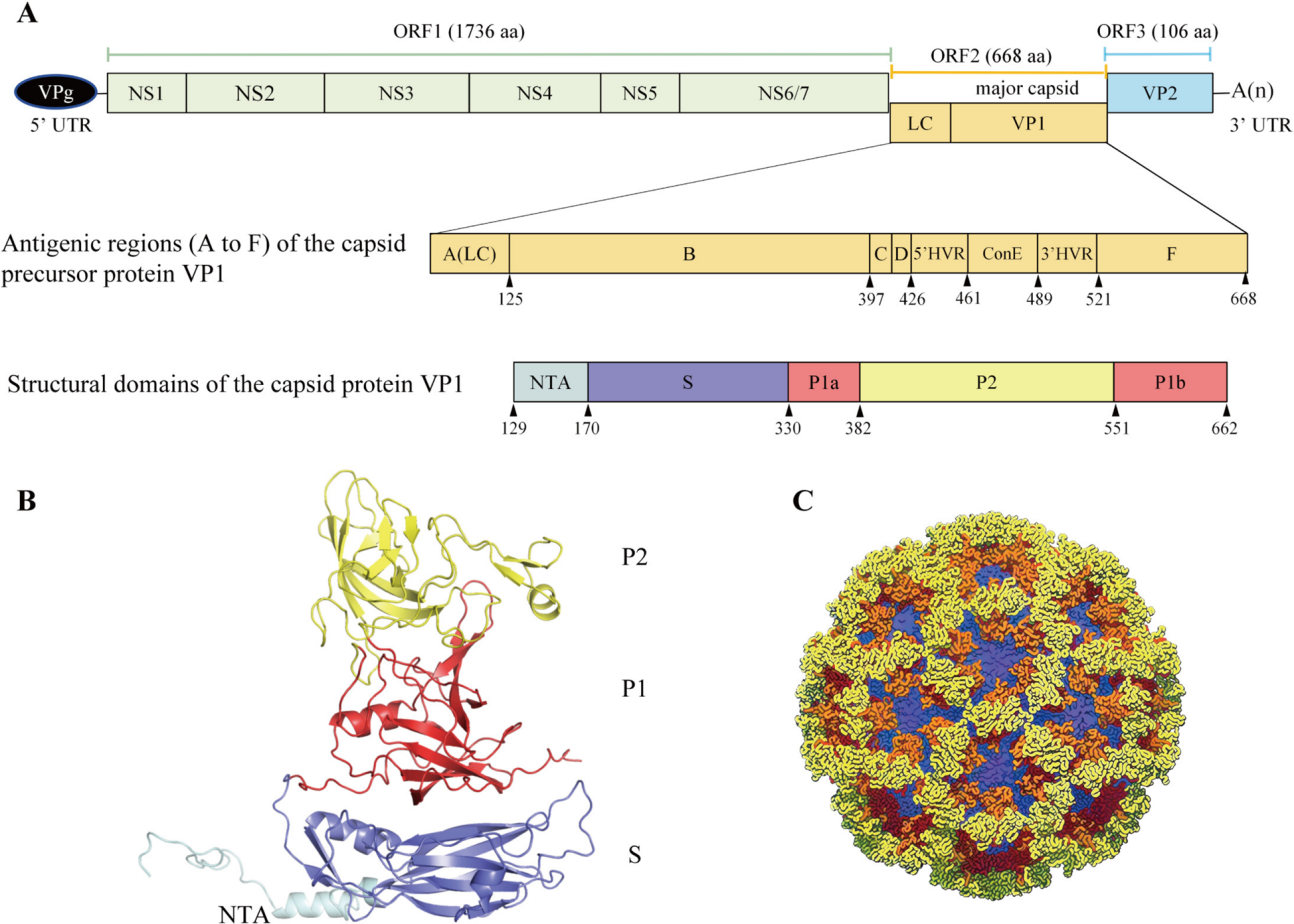
**Schematic representation of the feline calicivirus (FCV) genome and conserved calicivirus capsid architecture.** **A** Genomic organization of FCV, showing open reading frames (ORFs) 1–3, antigenic regions (A–F) within the VP1 capsid precursor, and structural domains of the mature VP1 capsid protein. **B** Ribbon diagram of the FCV VP1 capsid protein (Protein Data Bank [PDB] ID: 3M8L). **C** X-ray crystal structure of the Norwalk virus capsid [1], a norovirus representative of the Caliciviridae family, illustrating the conserved S-P1-P2 domain architecture. The S, P1, and P2 domains are colored blue, red, and yellow, respectively. LC, leader of the capsid protein; HVR, hypervariable region; ConE, central conserved region; NTA, N-terminal arm; S, shell domain; P, protruding domain comprising subdomains P1 (P1a and P1b) and P2.

The FCV genome is approximately 7.7 kb in length and contains three major open reading frames (ORFs) [4]. ORF1, located at the 5′ terminus, spans ~5.3 kb and encodes a polyprotein that is proteolytically cleaved into nonstructural proteins, including p5.6, p32, p39, p30, p13, and p76. Most of these nonstructural proteins are essential for viral translation and RNA replication [5–7]. ORF2 can be partitioned into six regions (A–F) on the basis of sequence conservation [8]. Region A encodes the leader capsid protein (LC); regions B, D, and F are relatively conserved, whereas C and E are hypervariable [9, 10]. Notably, E region harbors critical antigenic epitopes and mediates binding to the feline junctional adhesion molecule A (fJAM-A) receptor, whereas F region contains non-neutralizing epitopes [11–13]. ORF2 encodes a precursor protein (73–78 kDa) that is ultimately processed into the mature major capsid protein VP1 (~60 kDa). VP1 is a multifunctional protein that governs virion assembly, pathogenicity, and immunogenicity. Structurally, VP1 comprises three primary domains: an N-terminal arm (NTA) involved in capsid assembly [14], a shell domain (S) that encapsidates the viral RNA [15], and a protruding domain (P)—the most exposed region of the capsid—subdivided into P1 and P2 subdomains [2, 16] (Figure 1). Sequence variation in the *ORF2* gene underpins the classification of FCV strains into two genogroups, GI and GII, which differ by only three consistent amino acid substitutions: N377K, A539V, and G/P557S [17, 18]. To date, GII appears to consist exclusively of Asian isolates [17, 19, 20]. ORF3, located at the 3′ end of the genome, encodes the minor capsid protein VP2, which is indispensable for the assembly of infectious virions [21] and is proposed to form a portal-like complex that mediates viral genome delivery into host cells [22].

FCV exhibits high transmissibility among both domestic and wild felids [23], with kittens under 1 year of age being particularly susceptible. Infection with conventional FCV strains typically manifests as oral ulcerations, gingivostomatitis, pyrexia, anorexia, and ocular or nasal discharge, resulting in high morbidity but generally low mortality [24]. Acutely infected cats shed the virus predominantly in oculonasal secretions, although viral RNA can also be detected in blood, urine, and feces [25]. Since its first isolation in 1957 [26], FCV has become endemic in domestic cat populations worldwide [27–29]. In recent years, the continued emergence of highly virulent systemic feline calicivirus strains (VS-FCV) has raised significant concern. These variants cause virulent systemic disease (VSD), characterized by persistent high fever, facial and limb edema, necrotizing pancreatitis, hepatic necrosis, and high mortality rates [30–32]. Although vaccination remains the cornerstone of FCV prevention, its protective efficacy is increasingly compromised by extensive viral circulation and ongoing genetic evolution.

Here, we report the isolation and genetic characterization of 13 feline calicivirus strains from Jilin Province, China, between 2018 and 2024. These isolates display considerable phylogenetic diversity and antigenic heterogeneity, including variants exhibiting reduced susceptibility to vaccine-induced neutralization. Comparative analysis of two closely related yet antigenically distinct isolates, JL2103 and JL2104, revealed specific amino acid substitutions that may contribute to immune evasion. Collectively, our findings provide insights into the evolutionary dynamics of contemporary feline calicivirus under immune pressure and implicate specific residues as targets for rational vaccine design.

## Materials and methods

### Sample collection and reverse-transcription polymerase chain reaction (RT–PCR)/PCR detection

Between April 2018 and June 2024, oropharyngeal, ocular, and nasal swabs were collected from cats exhibiting clinical signs consistent with FCV infection, such as oral/lingual ulceration, ocular/nasal discharge, conjunctivitis, sneezing, and coughing, among others, at nine veterinary clinics in Jilin Province, China (six in Changchun city and three in Jilin city). Samples were included in the study if they were collected from cats meeting the clinical signs above, and if the swab sample volume was sufficient for both molecular detection (RT–PCR) and virus isolation. Samples were excluded if the volume was insufficient for both assays, or if the cat had received antiviral therapy (e.g., famciclovir, interferon) within 7 days prior to sampling. According to veterinary medical records, cats were categorized as “vaccinated” if they had received the complete course of the feline trivalent vaccine (which includes an inactivated FCV strain). Cats with no vaccination history or an incomplete vaccination schedule were categorized as “unvaccinated.”

Each swab was individually homogenized in phosphate-buffered saline (PBS) and centrifuged at 12000 *g* for 10 min at 4 °C. Total nucleic acids were extracted from the supernatant using a FastPure Viral DNA/RNA Mini Kit (Vazyme, China) following the manufacturer’s protocol. For primary FCV screening, a 260-base pair (bp) fragment of the *ORF1* gene was amplified using the PrimeScript™ One Step RT-PCR Kit version 2 (Takara, Japan) with FCV-specific primers (FCV-F/FCV-R). The thermal cycling conditions were as follows: reverse transcription at 50 °C for 30 min; initial denaturation at 95 °C for 5 min; 30 cycles of denaturation at 95 °C for 15 s, annealing at 55 °C for 15 s, and extension at 72 °C for 3 s; followed by a final extension at 72 °C for 10 min. Amplified products were visualized by agarose gel electrophoresis. To assess potential coinfections, all FCV-positive samples were further analyzed using 2× Rapid Taq Plus Master Mix (Dye Plus) (Vazyme, China) with pathogen-specific primer sets (Table 1). The PCR protocol consisted of the following steps: initial denaturation at 95 °C for 5 min; 30 cycles of denaturation at 95 °C for 30 s, annealing at 55 °C for 30 s, and extension at 72 °C for 1 min; and a final extension at 72 °C for 7 min.

**Table 1:** **Primers used in this study**

Name of primer	Primer sequence (5′ to 3′)	Target pathogen	Length of PCR product (bp)
FCV-F	GCTCAACCTGCGCTAACGTG	Feline calicivirus (FCV)	260
FCV-R	CCARTGCATTGGDGGVAC		
FPV-F	ATGGTTGGTGACTCTTTGTTT	Feline panleukopenia virus (FPV)	392
FPV-R	TACATTTGATTGACACTTCCC		
FHV-1-F	GACGTGGTGAATTATCAGC	Feline herpesvirus 1 (FHV-1)	287
FHV-1-R	CAACTAGATTTCCACCAGGA		
*M. felis*-F	TAGAAAGCAACGGCTAACTATGTG	*Mycoplasma felis* (*M. felis*)	504
*M. felis*-R	GCAGAAGATGTCAAGAGTGGGTAA		
*C. felis*-F	ATGAAAAAACTCTTGAAATCGG	*Chlamydia felis* (*C. felis*)	1094
*C. felis*-R	CAAGATTTTCTAGACTTCATTTTGTT		

### Virus isolation and identification

Supernatants from FCV-positive, noncoinfected samples were filtered through 0.22-μm syringe filters and inoculated onto confluent monolayers of F81 cells. Cells were maintained in Dulbecco’s modified Eagle medium (DMEM) supplemented with 5% fetal bovine serum at 37 °C in a 5% CO_2_ incubator until extensive cytopathic effect (CPE) was observed. Virus cultures were subjected to three freeze–thaw cycles, and the harvested lysates were purified by plaque assay. Viral isolates were confirmed by indirect immunofluorescence assay using guinea pig polyclonal antiserum against FCV VP1 as the primary antibody and Alexa Fluor 594-conjugated goat anti-guinea pig IgG (Yeasen, China) as the secondary antibody. Immunostained cells were examined by fluorescence microscopy.

### Amplification and sequence analysis of the *ORF2* gene

The complete *ORF2* genes of all 13 viral isolates were amplified using the primer pair FCV-F2 (5′-TGTGATGTGTTCGAAGTTTG-3′) and FCV-R2 (5ʹ-AATCAGGCCTAATATTGAAT-3ʹ). Reverse transcription–PCR was performed under the following conditions: reverse transcription at 50 °C for 30 min; initial denaturation at 95 °C for 5 min; 30 cycles of denaturation at 95 °C for 30 s, annealing at 55 °C for 30 s, extension at 72 °C for 1 min; and a final extension at 72 °C for 10 min. The amplified products were purified and cloned into the pMD19-T vector (Takara, Japan). Recombinant plasmids were sequenced by Sangon Biotech (Shanghai, China). The obtained* ORF2* nucleotide sequences and their deduced amino acid sequences were aligned with reference sequences retrieved from GenBank using SnapGene and MegAlign (Lasergene version 7.2, DNASTAR). Maximum-likelihood phylogenetic trees were inferred using MEGA11 (version 11.0), with branch support assessed by 1000 bootstrap replicates.

### Cross-neutralization assay

The vaccine antiserum was raised against Fel-O-Vax^®^ PCT (Zoetis), a commercially available trivalent inactivated vaccine widely used in China. Three healthy domestic cats aged 8–10 weeks were vaccinated with strict adherence to the manufacturer’s protocol. Blood samples were collected 21 days post-immunization, and sera were subsequently pooled within the group. Isolate-specific mouse antisera were prepared by immunizing groups of 6-week-old female BALB/c mice (*n* = 5 per group) with four distinct FCV field isolates (JL1907, JL1909, JL2103, and JL2104), respectively. Briefly, mice in each group were subcutaneously immunized with one of the β-propiolactone-inactivated isolates (1:3000 vol/vol; Serva, Germany) emulsified in Freund’s complete adjuvant, followed by two booster immunizations with Freund’s incomplete adjuvant at 21-day intervals. Sera were individually collected from each mouse and pooled within groups 21 days after the final booster immunization. For the cross-neutralization assay, serial twofold dilutions of antisera (vaccine antiserum or isolate-specific mouse antisera, starting at 1:4) were mixed with an equal volume of each of the 13 FCV isolates (200 50% tissue culture infectious dose [TCID_50_]) and incubated at 37 °C for 1 h. The serum–virus mixtures were then transferred to 96-well plates containing confluent F81 cell monolayers and incubated at 37 °C in a 5% CO_2_ atmosphere for 5 days. Virus neutralizing antibody (VNA) titer of each antiserum was defined as the reciprocal of the highest serum dilution that protected 50% of cell wells from visible virus-induced cytopathic effect, and titers were calculated via the Reed–Muench method. Each antiserum was tested in three independent cross-neutralization assays. Statistical analyses were performed using GraphPad Prism (version 9). Neutralization titers were subjected to log_2_-transformation before statistical analysis, and data presented in the figures and text represent the mean ± standard deviation (SD) of log_2_-transformed titers from three independent experiments.

### Structural mapping and interaction analysis of P2 domain variants between JL2103 and JL2104

The three-dimensional structures of the VP1 proteins of FCV-JL2103 and FCV-JL2104 were predicted using AlphaFold 3 via the AlphaFold Server [33]. The optimal structural models were selected according to the following confidence metrics: the predicted local distance difference test (pLDDT) score, where a value > 90 denotes high confidence, 70–90 indicates confident, 50–70 represents low confidence, and < 50 means very low confidence; and the predicted template modeling (pTM) score, where a value > 0.5 suggests consistency with the native structure and a score > 0.7 indicates high confidence [34]. Structural visualization and interaction analysis were performed using PyMOL (version 2.6, Schrödinger, LLC). Hydrogen bonds were identified using the built-in polar contacts function following this workflow: [A] → find → polar contacts → to other atoms in object. This command automatically detects all potential hydrogen bonds between the selected object and other atoms within the same object on the basis of PyMOL’s default geometric criteria, which are adapted from the DSSP algorithm [35] (donor–acceptor distance ≤ 3.6 Å, D-H-A angle 120–180°) to ensure analytical reliability. Salt bridges were identified by measuring the interatomic distances between oppositely charged side chains, with a distance cutoff set at ≤ 4.0 Å [36].

## Results

### Virus isolation and identification

**Figure 2:**
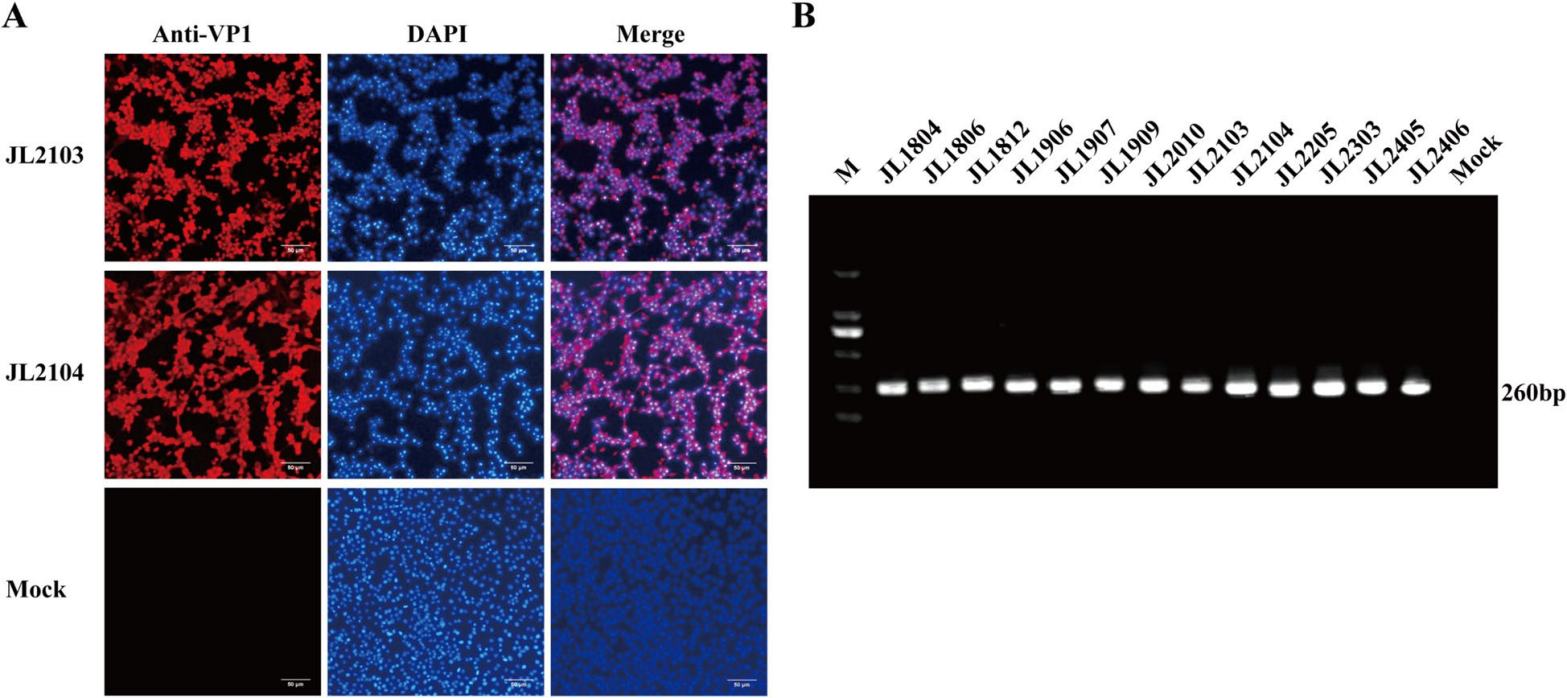
**Isolation and identification of 13 FCV isolates.** **A** Indirect immunofluorescence assay of F81 cells infected with FCV isolates JL2103 and JL2104 at 12 h post-infection. Cells were fixed with 4% paraformaldehyde and incubated with guinea pig polyclonal antiserum raised against VP1, followed by Alexa Fluor 594-conjugated goat anti–guinea pig IgG secondary antibody (red). Nuclei were counterstained with 4′,6-diamidino-2-phenylindole (DAPI; blue). Immunofluorescence results for the remaining 11 FCV isolates are provided in Additional file 1. **B** RT–PCR amplification with FCV-specific primers FCV-F and FCV-R using viral RNA from the 13 FCV isolates. M, Takara DNA Ladder 2000; lanes 1–13, ~260-bp amplicons; lane 14, mock control.

A total of 458 swabs were collected between April 2018 and June 2024. Of these, 127 samples tested positive for FCV by RT–PCR. Among these FCV-positive samples, 81 (63.8%) were coinfected with one or more other feline pathogens, including feline herpesvirus type 1 (FHV-1), feline panleukopenia virus (FPV), *Mycoplasma felis*, or *Chlamydia felis*, and were excluded from virus isolation. Virus isolation in F81 cells was performed on the remaining 46 FCV-positive samples, from which 13 FCV isolates were successfully obtained. A low isolation success rate (28.3%, 13/46) may be attributed to low viral loads and/or prolonged sample storage. Interestingly, three of these isolates were obtained from vaccinated cats. The clinical presentations and vaccination statuses of the 13 cats from which these isolates were obtained are summarized in Table 2. All isolates were confirmed as FCV by indirect immunofluorescence assay (Figure 2 and Additional file 1).

**Table 2:** **Epidemiological and clinical metadata for the 13 FCV isolates**

Isolate	Breed	Collection year	Geographic origin	Age (months)	Sex	Vaccination status	Clinical manifestations
JL1804	Chinese rural cat	2018	Changchun	9	Female	Unvaccinated	Nasal discharge
JL1806	American shorthair	2018	Changchun	5	Female	Vaccinated	Conjunctivitis, sneezing
JL1812	Garfield	2018	Jilin	3	Female	Unvaccinated	Oral ulceration, sneezing
JL1906	Chinese rural cat	2019	Changchun	9	Female	Unvaccinated	Anorexia, nasal discharge
JL1907	Hybrid	2019	Changchun	12	Male	Vaccinated	Oral ulceration
JL1909	Ginger cat	2020	Changchun	6	Female	Unvaccinated	Conjunctivitis, coughing
JL2010	Ginger cat	2020	Changchun	4	Male	Unvaccinated	Nasal discharge, sneezing
JL2103	Garfield	2021	Jilin	8	Female	Vaccinated	Conjunctivitis, nasal discharge
JL2104	British shorthair	2021	Jilin	2	Female	Unvaccinated	Ocular secretions, sneezing
JL2205	Ragdoll	2022	Changchun	7	Male	Unvaccinated	Conjunctivitis, sneezing
JL2303	Ragdoll	2023	Changchun	6	Female	Unvaccinated	Lingual ulceration
JL2405	Chinese rural cat	2024	Changchun	3	Male	Unvaccinated	Conjunctivitis, sneezing
JL2406	Hybrid	2024	Changchun	5	Male	Unvaccinated	Lingual ulceration

### Homology and phylogenetic analysis of FCV isolates

**Figure 3:**
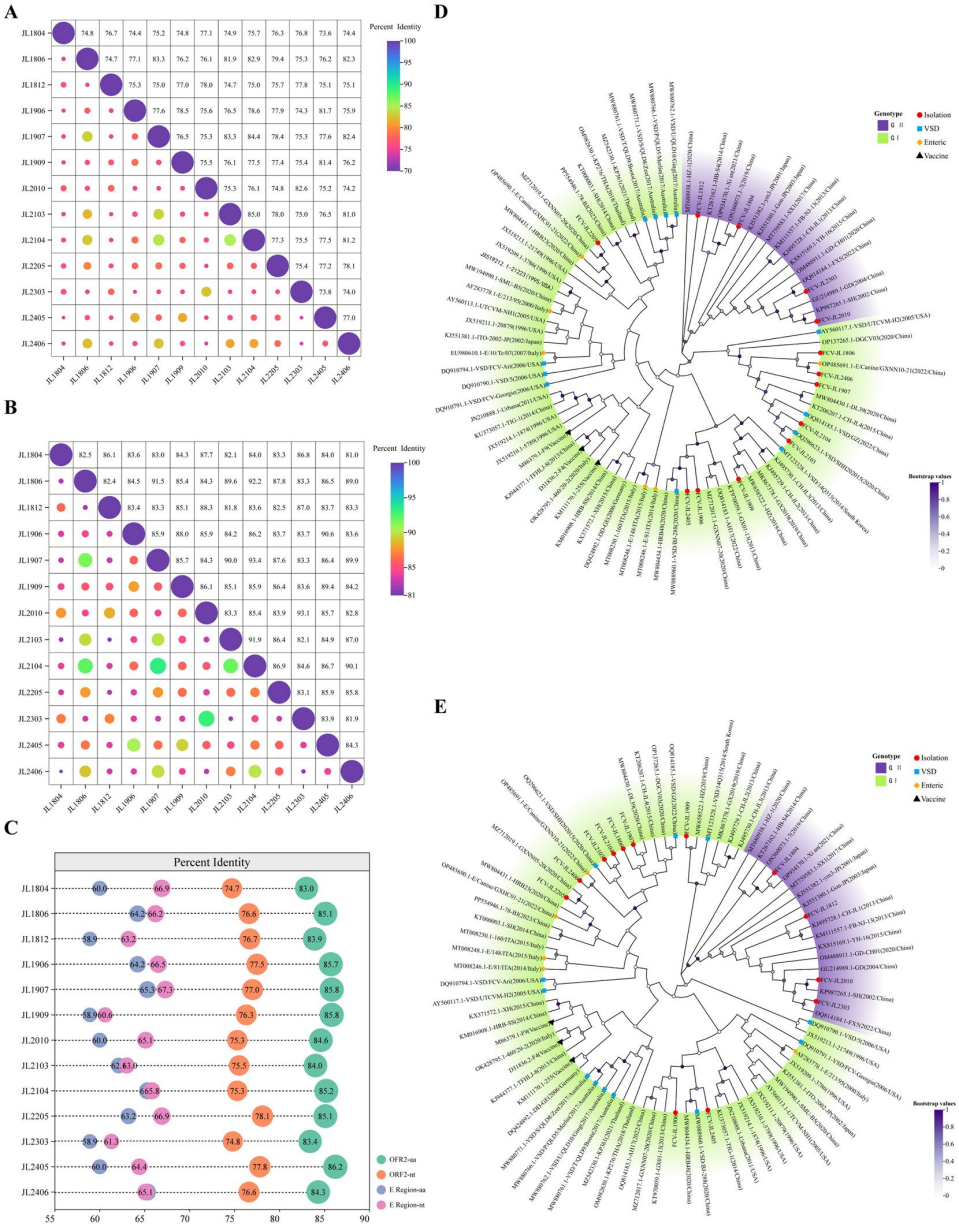
**Homology and phylogenetic analyses of 13 FCV isolates.** **A**, **B** Correlation heatmaps of pairwise nucleotide (**A**) and amino acid (**B**) identities among the 13 FCV isolates. Identity values are shown as circles on a continuous color scale; warmer colors and larger circles indicate higher sequence similarity. The numbers specify the exact identity percentages. **C** Lollipop plot of sequence identity between each FCV isolate and the FCV-255 vaccine strain. Circle color indicates the genomic region and sequence type: green, *ORF2* amino acid; orange, *ORF2* nucleotide; teal, E region amino acid; magenta, E region nucleotide. Circle size reflects the level of identity. Adjacent numbers represent the specific identity percentages. **D**, **E** Maximum-likelihood phylogenies based on complete *ORF2* nucleotide sequences (**D**) and deduced VP1 amino acid sequences (**E**), including the 13 FCV isolates from this study and 70 reference strains from GenBank. Symbols denote: red circles, isolates characterized in this study; black triangles, licensed vaccine strains (FCV-255, F4, F9); blue squares, virulent systemic disease (VSD)-associated strains; orange diamonds, enteric-origin FCV strains. The best-fit nucleotide substitution model is GTR + G + I, and the best-fit amino acid model is JTT + G + F. Branch support values were estimated from 1000 bootstrap replicates. Trees were visualized using ChiPlot [65].

The complete *ORF2 *sequences of the 13 FCV isolates ranged from 2007 to 2010 nucleotides (nt) in length, encoding VP1 proteins of 668–669 amino acids (aa). Six strains (JL1806, JL1907, JL2103, JL2104, JL2205, and JL2406) harbored a glycine insertion at residue 127 within the conserved B region, while JL1812 contained a serine insertion at residue 494 in E region. Pairwise alignment of the full-length *ORF2* sequences revealed substantial genetic diversity among the isolates, with nucleotide and amino acid identities ranging from 73.6% to 85.0% and 81.0% to 93.4%, respectively (Figure 3A, B). Notably, JL2104 shared the highest nucleotide identity (85.0%) with JL2103 and the highest amino acid identity (93.4%) with JL1907. In China, the commercial trivalent inactivated vaccine containing the FCV-255 strain, FHV-1 605 strain, and FPLV Cu-4 strain is widely used to prevent and control infections caused by FCV, FHV-1, and FPLV in cats. In this study, however, all isolates exhibited limited sequence homology to the commercial vaccine strain FCV-255 (Zoetis), sharing only 74.7–78.1% nucleotide and 83.0–86.2% amino acid identities across the entire *ORF2* (Figure 3C). This divergence was markedly greater in E region, where identities relative to the vaccine strain were as low as 60.6–67.3% at the nucleotide level and 58.9–65.3% at the amino acid level (Figure 3C). Collectively, these findings demonstrate extensive genetic heterogeneity in the *ORF2* gene among contemporary FCV isolates in Jilin Province, China.

Phylogenetic trees were reconstructed using the complete* ORF2* sequences from the 13 isolates and 70 reference strains. The nucleotide-based tree (Figure 3D) resolved FCV into two primary genogroups, GI and GII, containing nine and four isolates, respectively. Within GI, a well-supported subclade comprised five of the current isolates together with six previously reported Chinese strains. Specifically, isolates JL1907, JL2103, and JL2104 clustered closely with DL39 (Heilongjiang), JL4 (Jilin), and two virulent systemic disease (VSD)-associated strains from Shanghai (GZ and SHH202015). Isolates JL2406 and JL1806 grouped with GXNN10-21 (Guangdong) and DGCV03 (Guangxi), respectively. The remaining eight isolates were distributed across several distinct lineages. Notably, JL2406 clustered with GCNN10-21, and JL2205 grouped closely with GXHC01-21, both of which were previously detected in canine samples. Importantly, none of the 13 isolates clustered with the licensed vaccine strains FCV-255, F4, or F9, of which F4 and F9 are commercial vaccine strains abroad. The amino acid-based phylogeny (Figure 3E) exhibited a largely consistent topology, with minor topological discrepancies likely attributable to synonymous nucleotide substitutions. Overall, the 13 FCV isolates did not form province-specific monophyletic lineages but instead exhibited phylogenetic intermixing with strains from multiple geographic regions.

### Amino acid variation analysis of E region

**Figure 4:**
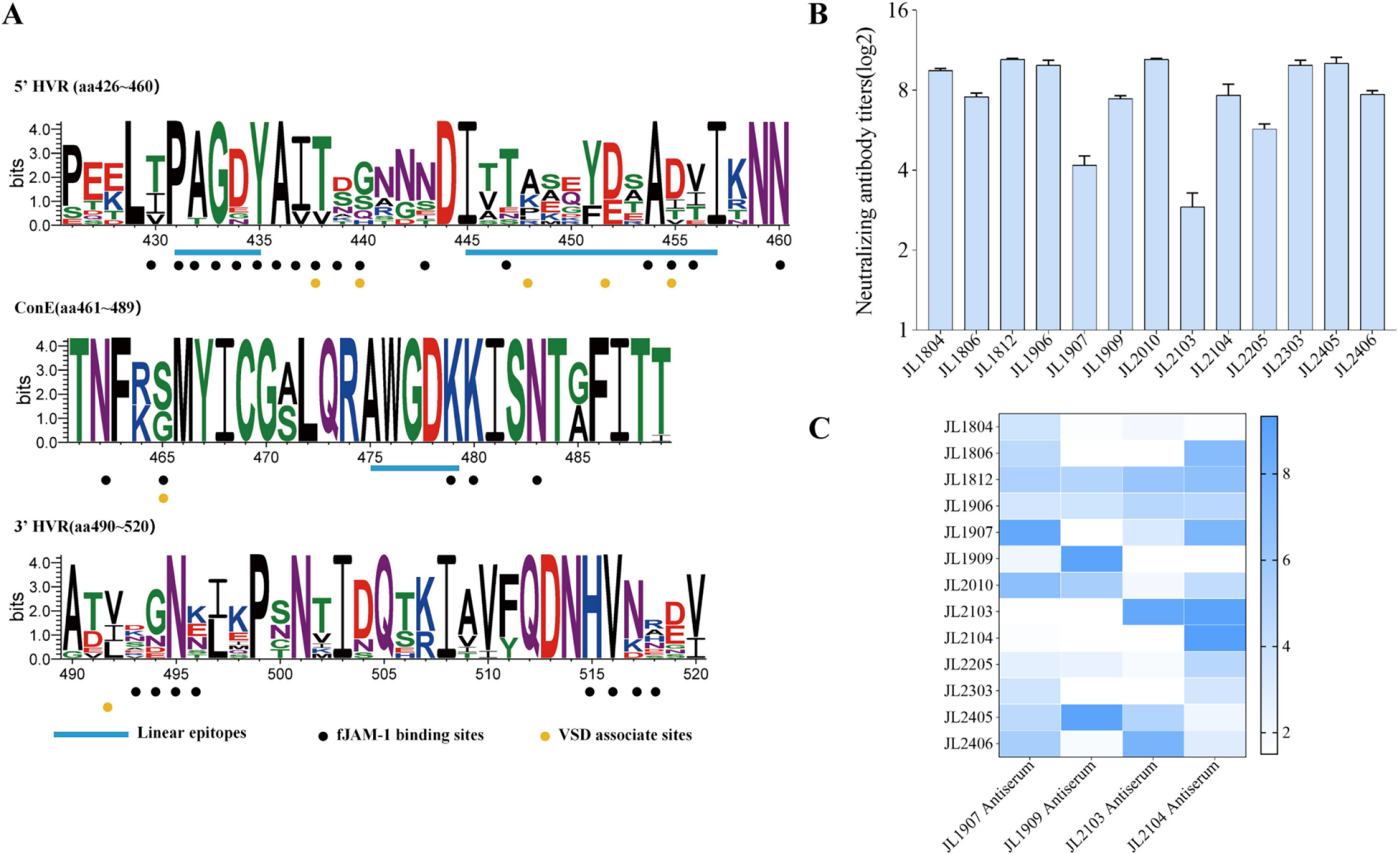
**Sequence variability in E region and neutralization profiles of the 13 FCV isolates.** **A** Sequence logo of E region showing amino acid variability among the 13 FCV isolates. Residue numbering is based on the full-length VP1 reference sequence of 668 amino acids. Sequences containing a single amino acid insertion (669 aa) were aligned to this reference framework. Blue bars indicate linear B cell epitopes; black circles mark residues involved in fJAM-A binding; orange circles denote residues associated with virulent systemic disease (VSD) strains. Hypervariable regions (5′-HVR-E and 3′-HVR-E) and the central conserved domain (ConE) are labeled. The logo was generated using WebLogo 3 [66]. **B** Neutralizing titers of FCV-255 vaccine antiserum against the 13 FCV isolates. Bars represent geometric mean neutralizing titers from three independent assays, with error bars indicating 95% confidence intervals. **C** Cross-neutralization heatmap of the 13 FCV isolates. Neutralizing titers for each antiserum (columns) against each isolate (rows) are displayed on a continuous color scale, with darker colors indicating higher neutralizing activity. Data represent geometric mean titers from three independent assays.

The E region of the major capsid protein VP1 contains critical antigenic epitopes and the fJAM-A receptor binding sites, making it a focal region in FCV research. Based on sequence conservation, this region is subdivided into two hypervariable segments, 5′-hypervariable region (HVR)-E and 3′-HVR-E, flanking a central conserved domain (ConE) [37, 38]. Consistent with this classification, the vast majority of amino acid substitutions among the 13 FCV isolates were confined to the 5′ and 3′ HVRs (Figure 4A). Two previously defined linear epitopes, residues 431–435 (PAGDY) [13] and 475–479 (AWGDK) [37], were largely conserved across all isolates, although single substitutions at positions 432 and 434 were detected in a subset of strains. In contrast, the neutralizing antibody epitope spanning residues 445–457 [37], including the core motif 445–451 (ITTANQY) [13], exhibited marked sequence heterogeneity. Comparative analysis further revealed amino acid variability at multiple residues previously implicated in fJAM-A binding [39] (Figure 4A). Of particular note, seven positions (438, 440, 448, 452, 455, 465, and 492) have been reported to differ between VSD and oral-respiratory disease (ORD) FCV strains [40]. Among the 13 FCV isolates, the G465S substitution was the most frequently observed change at these positions. Two isolates (JL2010 and JL2205) harbored four amino acid changes characteristic of VSD strains, whereas six isolates (JL1804, JL1806, JL1907, JL2103, JL2104, and JL2406) carried five or more substitutions typically observed in ORD strains (Table 3). Together, these observations demonstrate that the E region of the 13 FCV isolates displays pronounced sequence variation concentrated in known antigenic and receptor-interacting domains, with individual strains displaying amino acid signatures aligning either partially or predominantly with those previously described for VSD or ORD strains.

**Table 3:** **Amino acid variation at seven positions in E region associated with VSD or ORD FCV strains**

Genotype	Strains	Amino acids at seven VSD/ORD-associated sites						
		438	440	448	452	455	465	492
	VSD FCV	V	Q	K/R	E	T	S	V
	ORD FCV	T	G	A	D	D	G	V
G II	JL1804	T	G	A	D	I	G	L
G I	JL1806	T	G	A	D	D	**S**	I
G II	JL1812	T	**Q**	A	D	**T**	**S**	L
G I	JL1906	T	S	**K**	**E**	D	G	I
G I	JL1907	T	G	P	D	D	**S**	**V**
G I	JL1909	T	S	L	**E**	D	G	**V**
G II	JL2010	**V**	**Q**	**K**	D	V	**S**	L
G I	JL2103	T	G	P	D	D	**S**	**V**
G I	JL2104	T	G	A	D	D	**S**	**V**
G I	JL2205	T	G	**R**	**E**	**T**	G	**V**
G II	JL2303	**V**	H	A	D	I	**S**	L
G I	JL2405	T	S	**K**	**E**	D	G	I
G I	JL2406	T	G	A	D	D	**S**	**V**

Amino acid residues consistent with VSD were bolded. Residue numbering is based on the full-length VP1 reference sequence of 668 amino acids. Sequences containing a single amino acid insertion (669 aa) were aligned to this reference framework.

### Cross-neutralization assay

To assess the capacity of the widely deployed trivalent inactivated vaccine (Fel-O-Vax^®^ PCT, Zoetis) to neutralize antigenically diverse FCV field isolates, we performed cross-neutralization assays using antiserum raised against the vaccine. The vaccine antiserum potently neutralized the majority of the 13 FCV isolates. Six strains exhibited VNA titers exceeding 2^9^, and the highest mean titer reaching 2^10.44 ± 0.10^ (*n* = 3). Five additional isolates showed moderate neutralization, with VNA titers ranging from 2^5^ to 2^8^. In contrast, neutralizing activity against JL1907 and JL2103 was markedly reduced, with mean VNA titers of 2^4.19 ± 0.30^ and 2^2.91 ± 0.40^, respectively (Figure 4B). In parallel, antisera generated against JL1907, JL1909, JL2103, and JL2104 were tested against all 13 isolates. Homologous neutralization titers for these antisera were high, but cross-neutralization of heterologous isolates was generally limited. Notably, despite high nucleotide (83.3%) and amino acid (90.0%) identity in the *ORF2*-encoded VP1 protein between JL1907 and JL2103, their respective antisera exhibited minimal reciprocal cross-neutralization (Figure 4C). JL2104 shared the highest* ORF2* sequence identity with both JL2103 (85.0% nt, 91.9% aa) and JL1907 (84.4% nt, 93.4% aa). Nevertheless, it was neutralized only weakly by JL1907 antiserum (VNA = 2^1.66 ± 0.16^) and showed no detectable neutralization by JL2103 antiserum (VNA < 2^2^). In stark contrast, antiserum raised against JL2104 potently neutralized both JL1907 (VNA titer = 2^7.59 ± 0.16^) and JL2103 (VNA titer = 2^9 ± 0.34^) (Figure 4C). Collectively, these results show that vaccine-induced antiserum neutralized most isolates but failed to neutralize JL1907 and JL2103; among JL1907, JL2103, and JL2104, neutralization profiles were highly asymmetric, despite pairwise VP1 amino acid identities exceeding 90%.

### Interaction analysis of P2 domain variants between JL2103 and JL2104

**Figure 5:**
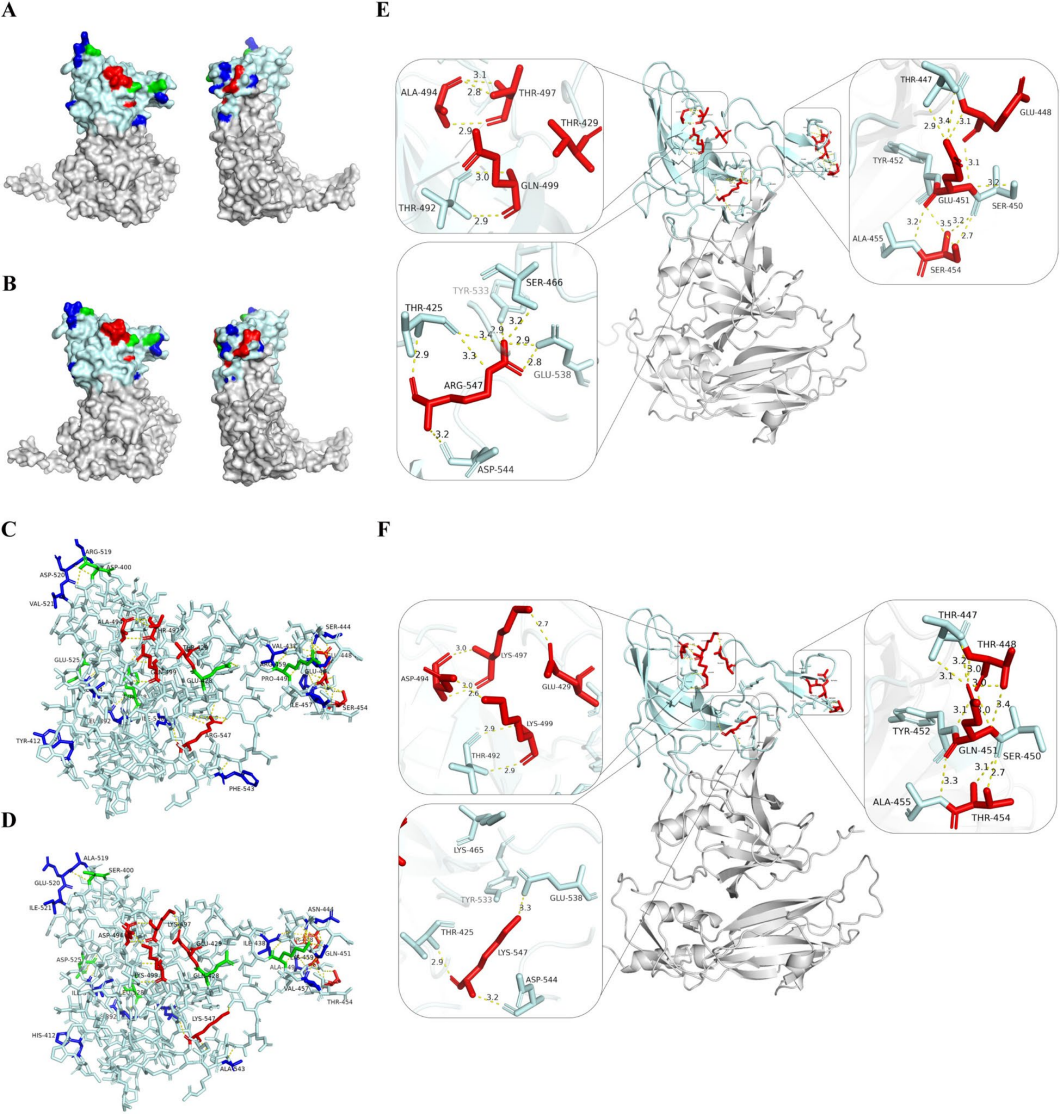
**Structural and interaction analysis of divergent residues in the P2 domain of JL2103 and JL2104.** **A**, **B** Spatial distribution of amino acid differences in the P2 domain between JL2103 (**A**) and JL2104 (**B**), shown in frontal and side views. **C**, **D** Noncovalent interaction networks involving variant residues in JL2103 (**C**) and JL2104 (**D**). **E**, **F** Representative variant residues in JL2103 (**E**) and JL2104 (**F**) exhibiting the most pronounced changes in hydrogen bonding patterns. The P2 domain backbone is rendered in pale cyan. Variant residues are color-coded as follows: blue, substitutions with no appreciable effect on local interactions; green, substitutions that form a single novel hydrogen bond with a neighboring residue; red, substitutions associated with extensive remodeling of interaction networks. Hydrogen bonds are depicted as dashed yellow lines. Structural models were predicted using AlphaFold3. Both models achieved a pTM score of 0.75, with pLDDT scores predominantly above 70. Interaction analyses were performed using PyMOL; residues with pLDDT < 70 were excluded to avoid potential artifacts arising from low-confidence regions.

A highly asymmetric neutralization pattern exists between JL2103 and JL2104: Antiserum against JL2103 failed to neutralize JL2104, whereas antiserum against JL2104 potently neutralized JL2103, despite these two strains sharing the highest VP1 amino acid identity among all 13 FCV isolates. Given that the P2 domain harbors both neutralizing antibody epitopes and the fJAM-A binding sites [38, 41], we focused our analysis on sequence variation within this region. Alignment of the P2 domains identified 25 amino acid differences between JL2103 and JL2104 (Figure 5). Of these, 12 substitutions did not appreciably alter local residue contacts, 5 introduced a single novel hydrogen bond with a neighboring residue, and the remaining 8 resulted in substantial reconfiguration of local interaction networks (Figure 5A–D).

Two regions exhibited especially complex changes involving multiple residues. As detailed in Figure 5E, JL2103 exhibited no hydrogen bonds at T429, three bonds between A494 and T497, and a Q499–T492 pair with two bonds. In contrast, JL2104 demonstrated contrasting patterns: one bond between E429 and K497, while D494 maintained two bonds with K497 (versus three in JL2103) and formed an additional connection to K499, which preserved the original two bonds with T492 (Figure 5F). Elsewhere, in JL2103, E448–E451 formed two hydrogen bonds, and E451 also engaged S450 and S454 via single hydrogen bonds each (Figure 5E); whereas in JL2104, the homologous K448–Q451 pair formed four hydrogen bonds but lacked interactions between Q451 and both T454 and S450 (Figure 5F). Another striking difference involved residue 547. In JL2103, R547 engaged in eight hydrogen bonds with five neighboring residues (T425 [3], S466 [1], Y533 [1], E538 [2], and D544 [1]) (Figure 5E). While, in JL2104, the K547 substitution drastically reduced this interaction network to just three hydrogen bonds (with T425, E538, and D544) (Figure 5F), representing a substantial destabilization of the local structure. Taken together, these results reveal that despite high VP1 sequence identity, specific amino acid substitutions in the P2 domain are associated with extensive remodeling of hydrogen bonding networks, which may account for the asymmetric neutralization pattern between JL2103 and JL2104.

## Discussion

Feline calicivirus (FCV) is a highly contagious, globally distributed pathogen in domestic cat populations. The error-prone replication of its single-stranded RNA genome drives rapid genetic diversification, which compromises diagnostic accuracy, vaccine efficacy, and antiviral interventions. Consequently, continuous surveillance of circulating FCV variants is essential to guide effective disease control measures. Although FCV has been extensively characterized across multiple regions of China, including Beijing [42], Shandong [43], Shanghai [44], Guangxi [18], and Guangdong [45], data from northeastern China, particularly Jilin Province, remain limited. In this study, we isolated and characterized 13 FCV strains from clinical samples collected in Jilin Province between 2018 and 2024, thereby addressing this knowledge gap. Genetic analysis of the major capsid gene (*ORF2*) revealed substantial sequence diversity among the isolates. Their distribution across multiple distinct phylogenetic clades (Figure 3E, F) further indicates considerable evolutionary divergence within the region. These findings illustrate the dynamic evolutionary characteristics of FCV in northeastern China, underscoring the region as a hotspot of viral diversification and enhancing our understanding of the spatial dynamics of FCV molecular evolution and population structure.

Although FCV is primarily recognized as a respiratory pathogen, it also exhibits enteric tropism. A recent surveillance study detected FCV in 25.9% of fecal samples from cats with diarrhea, frequently in coinfection with other enteric viruses, including feline panleukopenia virus (FPLV), feline coronavirus (FCoV), and feline astrovirus (FeAstV) [46]. While feline junctional adhesion molecule A (fJAM-A) serves as the primary cellular receptor for FCV entry, additional interactions with cell surface glycans (including binding to α2,6-linked sialic acids on MDCK cells) have also been reported [47]. This capacity for alternative receptor engagement offers a plausible mechanism for the reported isolation of FCV in diarrheic dogs [48–51]. Nevertheless, the epidemiological relevance of FCV in canine populations remains unclear, and further studies are required to determine whether these isolations represent incidental spillover or sustained cross-species transmission. Notably, although none of our 13 isolates originated from animals with diarrhea, several isolates collected over multiple years clustered phylogenetically with two FCV strains previously isolated in canine samples. This phylogenetic relatedness underscores the need for integrated, multi-host surveillance of enteric FCV circulation in northeastern China, particularly at the feline–canine interface.

The most severe manifestation of FCV infection is virulent systemic disease (VSD), a life-threatening syndrome marked by systemic inflammation, vascular endothelial damage, and multiorgan dysfunction [52]. In experimental infections, VSD-associated FCV strains consistently achieve higher replication titers in target tissues and elicit more pronounced viral shedding compared with classical respiratory strains [53, 54]. These strains also exhibit enhanced replication kinetics in vitro [55]. Since the emergence of VSD, significant efforts have been directed toward identifying genetic determinants underlying its enhanced pathogenicity. A recent hypothesis suggests that a specific constellation of seven amino acid substitutions in the VP1 capsid protein may contribute to the distinct virulence of VSD strains [40]. In our study, six isolates (JL1804, JL1806, JL1907, JL2103, JL2104, and JL2406) carried five or more substitutions of the seven VP1 substitutions associated with the oral respiratory disease (ORD) phenotype (Table 3). Five of these isolates clustered phylogenetically with well-characterized VSD strains. In contrast, JL2405 is closely related to VSD strains in the phylogeny but shares only two residues with the VSD-associated mutational profile. This observation challenges the proposed link between the seven-residue constellation and VSD virulence, in agreement with earlier studies that question its reliability as a virulence determinant [45, 56]. Given that the molecular mechanisms driving VSD pathogenesis remain incompletely understood, phenotypic characterization, including in vitro replication kinetics and in vivo challenge studies, currently provides the most robust means to differentiate VSD strains from ORD strains.

Although commercial FCV vaccines have reduced the incidence of clinical disease, breakthrough infections continue to occur in fully vaccinated cats [18, 45, 57]. While nonbiological factors (such as suboptimal vaccination protocols) may contribute, the predominant cause is likely antigenic drift driven by the adaptive evolution of FCV, enabling escape from vaccine-induced neutralizing antibodies. To assess antigenic relatedness between circulating strains and the vaccine strain, we performed virus neutralization assays using vaccine-induced antiserum against all 13 isolates. The antiserum effectively neutralized most strains, including four belonging to phylogenetic cluster GII (Figure 3), but exhibited markedly reduced neutralizing activity against JL1907 and JL2103 (VNA titers: 2^4.19 ± 0.30^ and 2^2.91 ± 0.40^, respectively). Both isolates were recovered from vaccinated cats, implying that current vaccines may confer limited cross-neutralization breadth against regional isolates. This inference requires confirmation through animal challenge studies. Our findings suggest that FCV immune evasion can arise from adaptive changes at a few key residues in the hypervariable E region of VP1. JL1907 and JL2103 evade vaccine-induced neutralization despite sharing > 90% VP1 amino acid identity with neutralization-sensitive strains such as JL2104. This indicates that antigenic divergence sufficient to impair cross-neutralization may be driven by localized substitutions rather than extensive sequence variation across the VP1 capsid protein.

The P2 subdomain of the capsid protein VP1 adopts a six-stranded β-barrel fold adorned with hypervariable surface loops [58], constituting the outermost and most exposed region of the virion. These loops serve as critical functional modules that mediate receptor engagement and harbor major antigenic determinants. Among the 25 amino acid differences between JL2103 and JL2104, residues 429, 448, 451, 454, 494, 497, 499, and 547 displayed the most pronounced perturbations in the local interaction network (Figure 5C, D). These residues localize to the hypervariable regions 5′-HVR-E, 3′-HVR-E, and the F region, respectively. Notably, within residues 445–451, JL2104 retains a sequence (ITTASQY, residues 446–452) closely resembling the the neutralizing epitope motif (ITTANQY [13]), whereas JL2103 exhibits a highly divergent variant sequence, ITEPSEY (residues 446–452). Molecular modeling indicated that the substitution at position 449 is conservative. In both JL2103 and JL2104, residue 449 maintains two hydrogen bonds with Y452, leaving the local backbone conformation unperturbed. In contrast, the divergent residues at positions 448 and 451, specifically E448T and E451Q, substantially reconfigured the local hydrogen-bonding network (Figure 5E, F). Several studies have reported motif variations at residues 445–451 in FCV isolates and suggested their potential association with immune escape [42, 45, 59, 60]. Our study provides a plausible structural basis for this antigenic mismatch. Another region exhibiting substantial differences in the local hydrogen-bonding network contains the divergent residues 429, 494, 497, and 499 between JL2103 and JL2104 (Figure 5E, F). Residues 494 and 497 correspond to reference positions 493 and 496 (Figure 4A), which were previously implicated in receptor engagement [39]. In many viruses, neutralizing epitopes frequently overlap receptor-binding sites, such as severe acute respiratory syndrome coronavirus 2 (SARS-CoV-2) [61], influenza virus [62], and measles virus [63]. Given this functional coupling between antigenicity and receptor binding, we propose that these divergent residues may contribute to the antigenic divergence observed between JL2103 and JL2104. This hypothesis is supported by the structural perturbations observed in our models. Additionally, the substitution at position 547 may represent another determinant of phenotypic differences and warrants functional characterization. Collectively, these findings suggest that minor amino acid substitutions at critical antigenic sites and receptor-proximal regions are sufficient to disrupt cross-neutralization between the highly homologous JL2103 and JL2104 strains. While previous studies have predominantly attributed vaccine failure and immune escape to substantial genetic divergence in VP1, especially within the hypervariable E region, between vaccine and field strains [15, 18, 64], our findings indicate that FCV can achieve antigenic escape without extensive genomic differences. However, we emphasize that these structural insights are based on computational models and require rigorous experimental validation, such as using FCV reverse genetics or pseudovirus systems, to establish causal links between specific residues and functional phenotypes.

In summary, we characterized the genetic and antigenic diversity of circulating feline calicivirus (FCV) strains in Jilin Province, China. Despite > 90% VP1 amino acid identity, some isolates showed markedly reduced cross-neutralization by vaccine-induced antibodies, associated with substitutions in hypervariable regions mapping to predicted neutralizing epitopes or receptor-proximal interfaces. Structural modeling implicates residues 429, 448, 451, 454, 494, 497, 499, and 547 in perturbing local interaction networks, suggesting a structural basis for antigenic divergence. We acknowledge several limitations in this study. The number of isolates was relatively modest and derived from a single Chinese province, which may not capture the full spectrum of FCV diversity across broader geographic scales. Our analysis focused exclusively on the *ORF2* gene; while this major capsid-encoding region is the primary determinant of antigenicity, contributions from other genomic regions cannot be excluded. Additionally, partial cross-neutralization assays were performed using mouse antisera rather than feline antisera, which may not fully recapitulate the feline immune response. Given genome-wide variation, causal roles require validation by site-directed mutagenesis in an isogenic background—ideally via FCV reverse genetics or pseudotyped viruses. Integrating these approaches with longitudinal surveillance and high-resolution structural characterization will be essential to delineate the molecular determinants of FCV antigenic evolution and guide rational design of broadly protective vaccines.

## Supplementary Information

**Additional file 1.** **Indirect immunofluorescence assay of F81 cells infected with the remaining 11 FCV isolates at 12 hours post-infection**. Cells were fixed with 4% paraformaldehyde and incubated with guinea pig polyclonal antiserum raised against VP1, followed by Alexa Fluor 594-conjugated goat anti–guinea pig IgG secondary antibody (red). Nuclei were counterstained with 4′,6-diamidino-2-phenylindole (DAPI; blue).

## Data Availability

The complete *ORF2* sequences of the 13 FCV isolates characterized in this study have been deposited to GenBank under accession nos. PZ148672–PZ148684.
