# Supplementary figures and images for: Localized adaptive evolution in VP1 drives antigenic divergence of feline calicivirus despite high sequence conservation
Source: Vet Res. 2026 May 26;57:85. doi: 10.1186/s13567-026-01778-y (PMC13214394; doi:10.1186/s13567-026-01778-y)

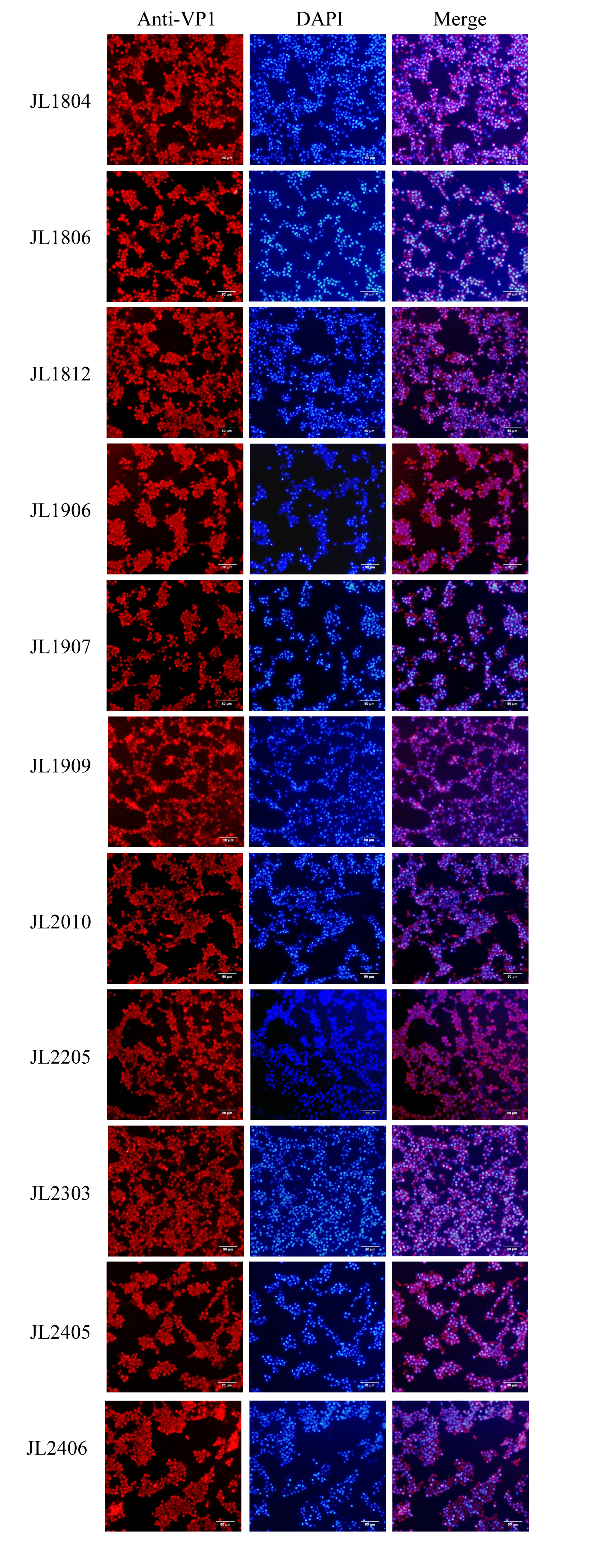

Supplement: Supplementary file 1 — Additional file 1. Indirect immunofluorescence assay of F81 cells infected with the remaining 11 FCV isolates at 12 hours post-infection. Cells were fixed with 4% paraformaldehyde and incubated with guinea pig polyclonal antiserum raised against VP1, followed by Alexa Fluor 594-conjugated goat anti–guinea pig IgG secondary antibody (red). Nuclei were counterstained with 4′,6-diamidino-2-phenylindole (DAPI; blue). [file 13567_2026_1778_MOESM1_ESM.tif]
